# Review of clinical characteristics and mortality outcomes in patients on maintenance hemodialysis during the Omicron surge: a single center experience

**DOI:** 10.1186/s12889-024-18999-5

**Published:** 2024-06-03

**Authors:** Yiyang Xue, Weiwei Feng, Ling Shi, Ning Cui, Wei Zhang, Junxiu Dong, Chunying Li, Jinjin Hu, Junjun Wei

**Affiliations:** 1 Health Science Center, Ningbo University, 818 Fenghua Rd, 315211 Ningbo, Zhejiang P. R. China; 2 Blood Purification Center, Ningbo Urol & Nephrol Hospital, 998 Qianhe Rd, 315100 Ningbo, Zhejiang P. R. China

**Keywords:** SARS-CoV-2, Omicron, Hemodialysis, Clinical characteristics

## Abstract

**Background:**

This hemodialysis center experienced the pandemic from December 2022 to January 2023. Therefore, we sought to describe the clinical characteristics and mortality outcomes in hemodialysis patients during this Omicron surge.

**Methods:**

According to whether they are infected, they are divided into two groups: SARS-CoV-2-positive and SARS-CoV-2-negative. The SARS-CoV-2-positive group was divided into a survival group and a non-survival group for comparison.

**Results:**

366 of 457 hemodialysis patients were infected with SARS-CoV-2. The most common symptoms observed were fever (43.2%) and cough (29.8%), Followed by diarrhea (1.4%). Hemodialysis patients with hypertension were more susceptible to SARS-CoV-2 infection. The lymphocyte count, serum creatinine, serum potassium, and serum phosphorus in the SARS-CoV-2-positive group were significantly lower than those in the SARS-CoV-2-negative group. The all-cause mortality rate for infection with SARS-CoV-2 was 5.2%. Only 7 of 366 SARS-CoV-2-positive patients were admitted to the intensive care unit, but 6 of them died. Intensive care unit hospitalization rates were significantly higher in the non-survival group compared with the survival group. White blood cells count, neutrophil count, C-reactive protein, AST, and D-dimer in the non-survival group were higher than those in the survival group. The lymphocyte count, hemoglobin concentration, serum creatinine, serum albumin, serum phosphorus and parathyroid hormone in the non-survival group were lower than those in the survival group. Age > 65 years, elevated C-reactive protein and AST are independent risk factors for death. Finally, no significant difference in vaccination status was found between the SARS-CoV-2-positive group and the negative group.

**Conclusions:**

Hemodialysis patients are at high risk for SARS-CoV-2 infection. Ensuring the adequacy of hemodialysis treatment and maintaining good physical condition of patients are the top priorities.

## Introduction

At the end of 2019, a new coronavirus named severe acute respiratory syndrome coronavirus 2 (SARS-CoV-2) emerged and quickly triggered an unusual viral pneumonia epidemic. Due to its high transmissibility, this novel coronavirus pneumonia, also known as coronavirus disease 2019 (COVID-19), has spread rapidly around the world [1]. This virus has mutated several times, among which the Omicron mutant strain will be the main circulating strain in China and even most parts of the world in 2022 [2]. According to a release from the Chinese Center for Disease Control and Prevention, from September 26, 2022 to January 23, 2023, China reported a total of 18,906 effective novel coronavirus genome sequences of local cases, all of which were Omicron mutant strains. From December 2022 to January 2023, the number of medical visits due to new coronavirus infection reached a peak in China, including outpatient medical treatment, general hospitalization and severe hospitalization [3].

Maintenance hemodialysis patients are a special category of patients during the novel coronavirus pandemic because they are at greater risk of exposure to infection than patients who are able to undergo home peritoneal dialysis. Although the pathogenicity of the Omicron variant is less than that of the previous strain, it is still accompanied by huge risks for patients receiving maintenance hemodialysis. The global prevalence of chronic kidney disease (CKD) is approximately 2-10% and varies over time in the same region [4–6]. The overall morbidity and mortality rates of CKD patients with SARS-CoV-2 are higher than those of individuals without kidney disease, including patients receiving dialysis [7–10].

In the past three years, there has not been a large-scale epidemic in the area where our center is located, and it only withstood greater epidemic challenges during the current Omicron surge. In the past, most studies were conducted during the epidemic period of Alpha, Beta and others, but there is a lack of research on the clinical characteristics of maintenance hemodialysis patients in the Omicron environment. Therefore, we retrospectively analyzed all patients who were registered in our center and received long-term maintenance hemodialysis from December 15, 2022 to February 22, 2023, a total of 457 patients. Our paper presents the clinical profiles and outcomes of maintenance hemodialysis patients with confirmed and undiagnosed suspected SARS-CoV-2 infection in our center, as well as survival and non-survival cases among maintenance hemodialysis patients with confirmed SARS-CoV-2 infection. clinical characteristics and established different profiles and outcomes between the two groups of patients.

## Patients and methods

### Study subjects

This retrospective study was conducted at the Hemodialysis Center of Urology and Nephrology Hospital, Ningbo, Zhejiang Province. We retrospectively analyzed the clinical results of 457 patients who were registered and maintained hemodialysis in our center from December 15, 2022 to February 22, 2023, and divided them into SARS-CoV-2 positive and SARS-CoV-2 negative two groups. In addition, based on the confirmed SARS-CoV-2 positivity, the patients were divided into a survival group and a non-survival group for comparison. The diagnostic criteria for patients included in the study refer to the “Diagnosis and Treatment Plan for Novel Coronavirus Infection (Tenth Edition)” issued by the National Health Commission of China [11]. The diagnostic criteria for suspected cases combined epidemiological history and clinical symptoms, and blood index tests, antigen-antibody and chest CT examinations were performed, so asymptomatic cases were discovered. All patients are over 18 years old. Patients receiving temporary hemodialysis were excluded. Follow-up ends at the end of the study or when the patient dies. All patients were informed and agreed to participate in this study and signed an informed consent form.

### Data collection

Data were collected from the hospital HIS system of the Hemodialysis Center of Urology and Nephrology Hospital in Ningbo, Zhejiang Province. Data extracted from registered patient medical records included demographic and clinical information, including age, gender, and chronic comorbidities. Data related to SARS-CoV-2 include clinical symptoms (fever, cough, myalgia, fatigue, taste/smell disorder, etc.), laboratory indicators, chest CT, whether to receive intensive care, and length of hospitalization. Data related to maintenance hemodialysis include hemodialysis age, dry weight, duration of dialysis (months), and dialysis urea clearance index (KT/V). Vaccination status of all patients receiving maintenance hemodialysis, including primary immunization, booster and secondary booster immunization.

### Statistical analysis

Statistical description: Normally distributed measurement data are described by mean ± standard deviation, non-normally distributed measurement data are described by median (interquartile range), and count data are described by frequency (percentage). Statistical analysis: Normally distributed measurement data were subjected to t test, and non-normally distributed measurement data were subjected to rank sum test. Count data were analyzed using chi-square test and Fisher’s exact probability method. Risk factor analysis used logistic regression analysis. *P* values < 0.05 were considered statistically significant.

## Results

### Epidemiological and clinical characteristics of all patients receiving maintenance hemodialysis

During the study period, a total of 457 people met the inclusion criteria, with an average age of 59.82 ± 14.13 years, 37.6% were female, including 366 SARS-CoV-2 positive patients and 91 SARS-CoV-2 negative patients.

Table 1 shows the comparison of epidemiological parameters, clinical symptoms, and hemodialysis-related data between SARS-CoV-2 positive and SARS-CoV-2 negative hemodialysis patients. The most common symptoms observed in hemodialysis patients after SARS-CoV-2 infection were fever (43.2%) and cough (29.8%), followed by diarrhea (1.4%). Other symptoms such as dyspnea, back pain, sore throat, headache, or taste/smell disorder are less common. However, the incidence of fatigue after infection with SARS-CoV-2 was reduced (0.5% vs. 4.4%). Patients with hypertension and hemodialysis showed statistical differences between the SARS-CoV-2 positive group and the negative group (*p* < 0.001). All patients receiving maintenance hemodialysis showed no statistically significant differences in dialysis-related data (hemodialysis age, dry weight, duration of dialysis (months), KT/V). Finally, no statistically significant difference in mortality was observed between the two groups (5.2% vs. 8.8%, *p* = 0.192). In addition, in terms of symptoms, fatigue showed completely opposite results to real-world observations (0.5% vs. 4.4%, *p* < 0.016).

**Table 1:** The baseline epidemiological and clinical characteristics of the patients

Variables	Total *n* (%)	SARS-CoV-2 (+) *n* (%)	SARS-CoV-2 (-) *n* (%)	*p*
Features	*N* = 457	*n* = 366	*n* = 91	
Age (years)	59.82 ± 14.13	59.27 ± 14.35	62.02 ± 13.02	0.097
< 40	41(9.0%)	37(10.1%)	4(4.4%)	0.217
40–65	251(54.9%)	200(54.6%)	51(56.0%)	
≥ 66	165(36.1%)	129(35.3%)	36(39.6%)	
Gender				
Male	285(62.4%)	230(62.8%)	55(60.4%)	0.672
Female	172(37.6%)	136(37.2%)	36(39.6%)	
Co-morbid disease				
Diabetes Mellitus	118(25.8%)	100(27.3%)	18(19.8%)	0.141
Coronary artery disease	12(2.6%)	10(2.7%)	2(2.2%)	0.775
Hypertension	447(97.8%)	364(99.5%)	83(91.2%)	< 0.001^*^
Heart failure	227(49.7%)	180(49.2%)	47(51.6%)	0.673
Cancer	31(6.8%)	27(7.4%)	4(4.4%)	0.311
Chronic obstructive lung disease	4(1.1%)	4(1.1%)	0(0.0%)	0.996
Symptoms				
Fever	197(43.1%)	158(43.2%)	39(42.9%)	0.957
Cough	137(30.0%)	109(29.8%)	28(30.8%)	0.854
Dyspnea	3(0.7%)	2(0.5%)	1(1.1%)	0.487
Back pain	3(0.7%)	2(0.5%)	1(1.1%)	0.487
Diarrhea	5(1.3%)	5(1.4%)	0(0.0%)	1.000
Fatigue	6(1.3%)	2(0.5%)	4(4.4%)	0.016^*^
Sore throat	5(1.1%)	3(0.8%)	2(2.2%)	0.260
Headache	1(0.2%)	0(0.0%)	1(1.1%)	0.358
Taste/smell disorder	1(0.2%)	1(0.3%)	0(0.0%)	0.362
HD-related indicators				
Hemodialysis age	52.50(42.25, 64.25)	52.25(42.00, 64.50)	54.00(44.50, 62.00)	0.649
Dry weight	58.40(51.84, 66.45)	58.34(51.68, 66.53)	59.20(52.18, 76.70)	0.924
Duration of dialysis (months)	59.93(17.39,104.84)	58.98(17.29,101.03)	75.93(19.38,120.10)	0.245
KT/V	1.47 ± 0.51	1.48 ± 0.53	1.46 ± 0.42	0.776
Vaccination status				
Fundamental immunity Booster immunization Secondary booster immunization	118(61.1%) 52(27.0%) 23(11.9%)	102(62.6%) 42(25.8%) 19(11.6%)	16(53.4%) 10(33.3%) 4(13.3%)	0.621
Mortality	27(5.9%)	19(5.2%)	8(8.8%)	0.192

* Values that reach statistical significance

In terms of mortality statistics, the all-cause mortality rate of 457 patients receiving maintenance hemodialysis was 5.9% (27/457), and the all-cause mortality rate of SARS-CoV-2-positive hemodialysis patients was 5.2% (19/366), while the all-cause mortality rate among SARS-CoV-2-negative hemodialysis patients was 8.8% (8/91). Through data analysis of all-cause mortality of the two groups of hemodialysis patients, no significant statistical difference was found (*p* = 0.192).

Table 2 shows the comparison of laboratory indicators between SARS-CoV-2-positive and SARS-CoV-2-negative hemodialysis patients. In terms of blood cell count, no significant difference was observed between the two groups in terms of the total number of white blood cells. It was only observed that the lymphocyte count in the SARS-CoV-2 positive group was lower than that in the SARS-CoV-2 negative group (0.85 ± 0.43 vs. 0.97 ± 0.47, *p* < 0.05). In the routine analysis of blood biochemistry, we found that the serum creatinine (Cr), serum potassium, serum phosphorus, and parathyroid hormone in the SARS-CoV-2 positive group were significantly lower than those in the SARS-CoV-2 negative group. Statistical significance (*p* < 0.05).

**Table 2:** Comparison of the laboratory findings of the patients

Variables	Total *n* (%)	SARS-CoV-2 (+) *n* (%)	SARS-CoV-2 (-) *n* (%)	*P*
Features	*N* = 457	*n* = 366	*n* = 91	
Complete blood count				
White blood cells (10^9^/L)	5.88 ± 2.50	5.87 ± 2.53	5.89 ± 2.39	0.957
<4	75(16.4%)	63(17.2%)	12(13.2%)	0.630
4–10	360(78.8%)	286(78.1%)	74(81.3%)	
>10	22(4.8%)	17(4.7%)	5(5.5%)	
Neutrophils (10^9^/L)	4.28 ± 2.26	4.31 ± 2.31	4.17 ± 2.04	0.579
Lymphocytes (10^9^/L)	0.87 ± 0.44	0.85 ± 0.43	0.97 ± 0.47	0.021^*^
Hemoglobin (g/L)	108.77 ± 16.58	108.07 ± 15.58	111.58 ± 19.98	0.120
<100	110(24.1%)	92(25.1%)	18(19.8%)	0.285
≥ 100	347(75.9%)	274(74.9%)	73(80.2%)	
Platelets (10^9^/L)	176.35 ± 71.31	178.55 ± 73.91	167.48 ± 59.28	0.186
C-reactive protein (mg/L)	2.90(0.50,14.30)	3.10(0.70,14.70)	2.20(0.50,9.50)	0.075
Creatinine (mg/dl)	836(571,1099)	824(540,1086)	920(649,1123)	0.043^*^
Ferritin	200.00(100.85,376.40)	209.50(81.60,375.90)	199.25(103.40,377.10)	0.996
ALT (U/L)	11(8,17)	11(8,17)	11(7,16)	0.451
AST (U/L)	14.5(11.0,19.0)	14.0(11.0,19.0)	15.0(11.0,21.0)	0.320
Albumin (g/L)	35.95 ± 4.88	35.80 ± 4.90	36.55 ± 4.79	0.194
<35	167(36.5%)	141(38.5%)	26(28.6%)	0.078
≥ 35	290(63.5%)	225(61.5%)	65(71.4%)	
Serum potassium (mmol/L)	4.54 ± 0.77	4.48 ± 0.74	4.80 ± 0.85	< 0.001^*^
Serum calcium (mmol/L)	2.18 ± 0.21	2.18 ± 0.21	2.17 ± 0.21	0.539
Blood phosphorus (mmol/L)	1.39 ± 0.50	1.34 ± 0.48	1.60 ± 0.55	< 0.001^*^
Parathyroid hormone (pg/ml)	303.00(177.93,464.33)	298.60(177.25,446.50)	352.20(180.50,581.40)	0.135
<150	93(20.3%)	72(19.7%)	21(23.1%)	0.048^*^
150–600	292(63.9%)	243(66.4%)	49(53.8%)	
>600	72(15.8%)	51(13.9%)	21(23.1%)	
D-dimer (mg/L)	331 (187,793)	328(185,7100)	360(209,1019)	0.509
<400	123(60.6%)	104(61.9%)	19(54.3%)	0.401
≥ 400	80(39.4%)	64(38.1%)	16(45.7%)	

* Values that reach statistical significance

During the follow-up period, we counted the vaccination status of all hemodialysis patients (Table 1). The vaccination rate for the first basic vaccination was the highest (61.1%), the booster vaccination rate dropped to 27.0%, and the second booster vaccination needle is only 11.9%. At the same time, no significant difference in vaccination status was found between the SARS-CoV-2 positive group and the negative group.

### Comparison of clinical characteristics and laboratory indicators according to mortality among all SARS-CoV-2-positive hemodialysis patients

Among the 366 SARS-CoV-2-positive patients, 19 died due to various causes including infection during hospitalization (5.2%), and the remaining 347 patients survived.

Table 3 shows the comparison of demographic and laboratory data between the two groups. Age < 65 years showed significant differences between the survival group and the non-survival group (*p* < 0.001), and age was an independent risk factor for death (Table 5). Secondly, we also found that there was a statistical difference between the two groups in terms of age at starting dialysis (*p* < 0.001), that is, the older the age at starting dialysis, the higher the mortality rate. In the comparison of comorbidities, we found that the comorbidity rates of cardiovascular disease, hypertension, and heart failure in the non-survival group were significantly higher than those in the survival group. Only 7 of the 366 SARS-CoV-2-positive patients were admitted to the intensive care unit (ICU), accounting for 1.9%. Compared with the survival group, ICU rate was significantly higher in the non-survival group (0.3% vs. 31.6%, *p* < 0.001). As shown in Table 4, we also found that the total number of white blood cells, neutrophil count, C-reactive protein, AST, and D-dimer in the non-survival group were higher than those in the survival group. Among them, C-reactive protein and AST are independent risk factors for death in infected patients (Table 5). Not only that, we also found that the lymphocyte count, hemoglobin concentration, blood creatinine, serum albumin, blood phosphorus, and parathyroid hormone indicators in the non-survival group were lower than those in the survival group.

**Table 3:** Clinical characteristics of SARS-CoV-2 positive hemodialysis patients by mortality

Variables	Total *n*(%)	Mortality		*p*
		Survivors *n*(%)	Nonsurvivors *n*(%)	
Features	*N* = 366	*n* = 347	*N* = 19	
Age(years)	59(49,70)	58(48,69)	75(67,81)	**< 0.001** ^*^
<65	230(62.8%)	226(65.1%)	4(21.1%)	**< 0.001** ^*^
≥65	136(37.2%)	121(34.9%)	15(78.9%)	
Gender				
Male	230(62.8%)	219(63.1%)	11(57.9%)	0.647
Female	136(37.2%)	128(36.9%)	8(42.1%)	
Co-morbid disease				
Diabetes Mellitus	97(26.5%)	91(26.2%)	6(31.6%)	0.607
Coronary artery disease	7(1.9%)	3(0.9%)	4(21.1%)	**< 0.001** ^*^
Hypertension	348(95.1%)	336(96.8%)	12(63.2%)	**< 0.001** ^*^
Heart failure	175(47.8%)	160(46.1%)	15(78.9%)	**0.005** ^*^
Cancer	26(7.1%)	25(7.2%)	1(5.3%)	1.000
Chronic lung disease	4(1.1%)	3(0.9%)	1(5.3%)	0.193
HD-related indicators				
Hemodialysis age	53.00(43.00, 65.00)	52.00(42.00, 64.00)	68.00(62.00, 78.00)	**< 0.001** ^*^
Dry weight	58.34(51.68, 66.53)	58.40(51.69, 66.88)	57.40(49.10, 62.80)	0.363
Duration of dialysis (months)	59.92(17.56, 102.53)	58.93(17.53, 102.50)	76.60(24.63, 118.60)	0.464
KT/V	1.47 ± 0.53	1.48 ± 0.53	1.37 ± 0.36	0.490
PCR				
Negative	100(28.6%)	100(30.2%)	0(0%)	**0.005** ^*^
Positive	250(71.4%)	231(69.8%)	19(100%)	
Chest CT				
Normal	27(12.4%)	26(12.7%)	1(7.7%)	0.194
Mild	105(48.4%)	101(49.5%)	4(30.8%)	
Moderate	81(37.3%)	74(36.3%)	7(53.8%)	
Severe	4(1.8%)	3(1.5%)	1(7.7%)	
Intensive care follow-up				
No	359(98.1%)	346(99.7%)	13(68.4%)	**< 0.001** ^*^
Yes	7(1.9%)	1(0.3%)	6(31.6%)	
Lenght of stay in hospital	10.0(5.0, 17.0)	10.0(5.0, 17.0)	11.0(5.5, 23.0)	0.473

* Values that reach statistical significance

**Table 4:** Laboratory findings of SARS-CoV-2 positive hemodialysis patients by mortality

Variables	Total *n*(%)	Mortality		*p*
		Survivors *n*(%)	Nonsurvivors *n*(%)	
Features	*N* = 366	*n* = 347	*n* = 19	
Complete blood count				
White blood cells (10^9^/L)	5.87 ± 2.53	5.80 ± 2.14	7.27 ± 6.30	**0.013** ^*^
<4	63(17.2%)	58(16.7%)	5(26.3%)	0.128
4–10	286(78.1%)	274(79.0%)	12(63.2%)	
>10	17(4.7%)	15(4.3%)	2(10.5%)	
Neutrophils (10^9^/L)	4.31 ± 2.31	4.21 ± 1.91	6.16 ± 5.82	**< 0.001** ^*^
Lymphocytes (10^9^/L)	0.85 ± 0.43	0.86 ± 0.42	0.53 ± 0.45	**0.005** ^*^
Hemoglobin (g/L)	108.07 ± 15.58	108.57 ± 14.36	98.90 ± 29.31	**0.008** ^*^
<100	92(25.1%)	82(23.6%)	10(52.6%)	**0.010** ^*^
≥ 100	274(74.9%)	265(76.4%)	9(47.4%)	
Platelets (10^9^/L)	178.55 ± 73.91	179.79 ± 72.24	155.95 ± 99.35	0.171
C-reactive protein (mg/L)	5.20(2.50, 20.90)	4.60(2.35, 17.00)	59.05(3.43, 97.20)	**0.001** ^*^
Creatinine (mg/dl)	824.50(540.00,1091.25)	831.00(576.00,1092.00)	387.00(273.00,858.00)	**< 0.001** ^*^
Ferritin	200.00(105.98, 387.00)	200.00(104.40, 389.10)	214.70(106.50, 383.80)	0.860
ALT (U/L)	11.00(8.00, 17.00)	11.00(8.00,17.00)	11.00(5.00, 26.00)	0.880
AST (U/L)	14.00(11.00, 19.00)	14.00(11.00,18.00)	19.00(11.00, 29.00)	**0.025** ^*^
Albumin (g/L)	35.85 ± 4.89	36.18 ± 4.64	29.86 ± 5.48	**< 0.001** ^*^
<35	140(38.3%)	125(36%)	15(78.9%)	**< 0.001** ^*^
≥ 35	226(61.7%)	222(64%)	4(21.1%)	
Serum potassium (mmol/L)	4.47 ± 0.74	4.49 ± 0.73	4.17 ± 0.75	0.081
Serum calcium (mmol/L)	2.18 ± 0.21	2.18 ± 0.20	2.24 ± 0.26	0.188
Blood phosphorus (mmol/L)	1.34 ± 0.48	1.36 ± 0.48	1.02 ± 0.38	**0.002** ^*^
Parathyroid hormone (pg/ml)	299.40(177.00,446.80)	300.25(181.70,456.15)	219.40(97.50,359.20)	**0.045** ^*^
<150	71(19.6%)	63(18.3%)	8(42.1%)	0.051
150–600	241(66.4%)	231(67.2%)	10(52.6%)	
>600	51(14.0%)	50(14.5%)	1(5.3%)	
D-dimer (mg/L)	324.00(184.00,718.00)	305.00(177.00,612.00)	1017.50(363.25,1093.50)	**0.006** ^*^
<400	105(61.4%)	101(65.2%)	4(25%)	**0.002** ^*^
≥ 400	66(38.6%)	54(34.8%)	12(75%)	

* Values that reach statistical significance

**Table 5:** Logistic regression analysis of influencing factors of death

Variables	B	SE	Wald	*P*	OR	OR(L)	OR(U)
Age	0.287	0.103	7.771	0.005^*^	1.333	1.089	1.631
C-reactive protein	0.021	0.009	5.457	0.019^*^	1.021	1.003	1.039
AST	0.043	0.018	5.791	0.016^*^	1.043	1.008	1.080

* The multi-factor influencing factor analysis adopts logistic regression and includes all indicators with single factor *P* < 0.15. The analysis result *P* < 0.05 is an independent influencing factor, OR value < 1 is a protective factor, and OR > 1 is a risk factor

## Discussion

Experience since the COVID-19 pandemic has shown that patients receiving maintenance hemodialysis are at higher risk of severe SARS-CoV-2 infection and severe consequences compared with other groups, as this group must undergo treatment 2–3 times a week There are also disadvantages associated with traveling between the hospital and home, such as relatively older age, multiple comorbidities, and suppressed immune systems [12]. Although previous experience has told us that SARS-CoV-2 infection seriously threatens the health of hemodialysis patients [13], most of these studies were conducted in the early stages of the pandemic. However, in this central area, due to effective control policies, there has never been a large-scale outbreak or an epidemic affecting a large number of people. Only during the Omicron surge period from mid-December 2022 to February 2023 did more infection cases occur [14], and the relevant research information on Omicron mutant strains is limited. Therefore, we aimed to add some experience to this small information base by describing the susceptibility characteristics, clinical manifestations, and short-term outcomes of SARS-CoV-2 infection in hemodialysis patients during this period, and analyzing mortality and influencing factors.

### Comorbidities and clinical symptoms

Previous studies have shown that older patients with comorbidities are at higher risk of infection and fatal outcomes [15, 16], and this situation is of greater concern in hemodialysis patients [17, 18]. Studies have confirmed that patients with hypertension are at a higher risk of infection than other groups because these patients often have higher levels of angiotensin-converting enzyme 2 (ACE2), which facilitates infection with SARS-CoV-2 [19]. The results of our study strongly support this view. We found that hemodialysis patients with hypertension are more susceptible to SARS-CoV-2 virus infection (*p* < 0.001). Not only that, certain comorbid factors such as hypertension, cardiovascular disease, heart failure and mortality showed statistically significant differences. In our study, comorbidities such as chronic lung disease and diabetes, which have received much attention in the past, had no significant impact on susceptibility and mortality.

Our study found that the most common symptoms observed after SARS-CoV-2 infection were fever (43.2%) and cough (29.8%), and these clinical characteristics are consistent with most studies [15, 16, 20–22]. Other symptoms, such as dyspnea, back pain, sore throat, headache or taste/smell disorder, occur in a much smaller proportion. Overall, our study found that the incidence of fever (43.2% vs. 42.9%) and cough (29.8% vs. 30.8%) in hemodialysis patients infected with SARS-CoV-2 was basically similar to that in uninfected people. Fatigue (0.5%) and diarrhea (1.4%) were rare manifestations in our study, however, studies have found that fatigue and gastrointestinal symptoms occur in high rates in hemodialysis patients after SARS-CoV-2 infection [23]. A study conducted by Wang et al. at Wuhan Zhongnan Hospital on 5 hemodialysis patients at the end of 2019 also reported that diarrhea (80%) was the most common manifestation [24]. Several cross-sectional studies have stated that the occurrence of fatigue in hemodialysis patients is related to sleep, living environment, and treatment conditions, and that elderly hemodialysis patients are prone to more severe fatigue [25, 26]. Although the correlation with SARS-CoV-2 has not been demonstrated, we speculate that the symptoms may be atypical due to the complex and variable clinical manifestations of SARS-CoV-2 infection. Therefore, relying solely on symptoms to make a diagnosis is blind and should be taken seriously.

### Laboratory indicators

Our study also found that in infected patients, in addition to common lymphopenia, increases in leukocytes, neutrophils, CRP, AST and D-dimer were associated with severe disease and death outcomes (*p* < 0.05). This aspect is basically consistent with previous reports [27–29]. Lymphopenia is very common in patients with common infections or deaths, which may be related to direct viral infection that consumes immune cells or induces inflammatory cascades [30–33]. In patients who died, we observed significant increases in white blood cells, neutrophils, and CRP. Some researchers have speculated on this phenomenon and pointed out that since bacterial co-infection is more common in severe patients than ordinary patients, it is reasonable to suspect that bacterial co-infection will aggravate the condition and affect the laboratory indicators [21]. CRP is also an independent risk factor for disease progression and death [34, 35]. This view was also proven in our study (Table 5). Compared with the non-survivors, hemoglobin concentration, albumin, blood phosphorus and parathyroid hormone remained at higher levels in the survivors (*p* < 0.05). Some studies have reported that the prevalence of hypophosphatemia is particularly significant among patients with end-stage renal disease infected with SARS-CoV-2 [36]. Some studies have shown that hypophosphatemia may be associated with increased mortality in critically ill patients [37]. The possible reasons for the imbalance of phosphate levels in hemodialysis patients are more complicated. Most of them are due to insufficient nutritional intake, especially in hospitalized patients. At the same time, patients with end-stage renal disease have impaired nutrient absorption due to primary diseases. Secondly, patients with SARS-CoV-2, especially those with respiratory distress, are often complicated by respiratory alkalosis, which further leads to a decrease in blood phosphate levels. In addition, vitamin D deficiency and obesity are also possible causes [38]. One study documented disorders in blood phosphorus, blood calcium, and parathyroid hormone in patients infected with SARS-CoV-2. The results indicate that the SARS-CoV-2 virus may disturb the balance, and SARS-CoV-2 has been confirmed to be a potential cause of hypoparathyroidism [39]. Serum potassium was only found to be statistically significant in the comparison between the SARS-CoV-2 positive group and the negative group in hemodialysis patients (Table 2). Although the indicators decreased in the positive group, they were still within the normal range. It is speculated that it may be related to the activation of renin-angiotensin-aldosterone (RAS) after infection with SARS-CoV-2 [40].

Studies have confirmed that infection with SARS-CoV-2 can induce a massive thrombotic state [41–43]. We have also observed this phenomenon, and clinicians at our dialysis center found that even when receiving anticoagulant doses (1000-3000IU) of low molecular weight heparin, some patients still found that intravenous pot dialyzer tubing developed during dialysis. There was coagulation in the patient, and our doctors intervened promptly to avoid a more dangerous event. A single-center retrospective study reported that despite the use of more anticoagulants, hemodialysis patients infected with SARS-CoV-2 are still at high risk of dialysis circuit coagulation [44]. Although our study results showed no significant difference in D-dimer changes between uninfected and infected hemodialysis patients, we then found that D-dimer increased in the comparison between the non-survivors and the survivors. There is significant statistical significance (*p* = 0.006). A recently published report reached a similar conclusion, showing the non-survivors had higher D-dimer levels compared with those who survived [45]. We also observed an increase in AST in the non-survivors (*p* < 0.05). Combined with the above changes in D-dimer, we suspect that SARS-CoV-2 is very likely to damage the myocardium and blood vessels. We found that in the comparison of infected and uninfected hemodialysis patients, there was basically no difference in liver function and albumin indicators. This may be in the early stages. Once the disease worsens or even leads to death, there is a significant difference between the increase in AST and the decrease in albumin. Among them, elevated AST is an independent risk factor for death (Table 5).

### Discussion on the impact of hemodialysis adequacy, nutritional status and age factors on clinical outcomes

Notably, our study also found that higher serum creatinine was associated with better survival outcome. Although for patients who receive maintenance hemodialysis for a long time, serum creatinine is often affected by the time and frequency of hemodialysis, but it can still be used to evaluate the patient’s nutritional status [46]. We have reason to speculate that higher serum creatinine reflects, to a certain extent, patients’ greater muscle mass, greater exercise capacity, and better nutritional conditions. Patients registered in our center and receiving long-term maintenance hemodialysis performed well in various indicators reflecting the body, such as KT/V, hemoglobin concentration, albumin, blood calcium, and blood phosphorus, all maintained at high levels (Table 3). To some extent, this reflects the nutritional status of the patient and the adequacy of hemodialysis. It is statistically significant that the hemoglobin, albumin, and blood phosphorus of deceased patients were significantly lower than those of surviving patients (*p* < 0.01). Studies have shown that hemoglobin concentration, serum albumin, and serum phosphorus are negatively correlated with mortality [37, 47, 48]. Finally, we boldly speculate that hemodialysis patients with better basic physical conditions will show stronger tolerance and better clinical outcomes during the surge of Omicron.

In the early stages of the SARS-CoV-2 epidemic in the past, many research centers had already considered age ≥ 65 years as one of the adverse prognostic factors [21, 49]. Even now when the Omicron strain is the main circulating strain, our study concluded that age is still an independent risk factor for death (Table 5). Age ≥ 65 years was associated with higher mortality (*p* < 0.001). Among dialysis-related indicators, although no difference between the two groups was observed in the comparison of duration of disorder, hemodialysis age was significantly different between survivors and non-survivors (*p* < 0.001), which showed that it was related to the age factor. Consistent results. Therefore, we concluded that older age (≥ 65 years or hemodialysis age) is associated with higher mortality.

### Vaccines and mortality

Up to now, data on immune cell responses following vaccination in patients on maintenance hemodialysis are limited. Most studies indicate that the vaccine response rate in the dialysis population is lower than expected and that the relative risk is higher in the dialysis population compared with the general population [50–54]. In our study, we asked 413 registered dialysis patients about their vaccination status. The results show that the majority (61.1%) received one dose, 27% received two doses, and only 11.9% received three doses (Table 1). And no significant difference was observed in the comparison between infected and uninfected patients (*p* = 0.621). We analyze that the reasons may be as follows: the vaccines administered to hemodialysis patients in our center are mainly divided into the first dose of adenovirus vector vaccine, the second dose of inactivated vaccine, and the third dose of recombinant protein vaccine. These types of vaccines have certain Due to the lag, we speculate that the mutation process of the virus may be much faster than the development and update of vaccines. Therefore, during the Omicron surge period, the protective effect of these vaccines developed against previous strains will be lower than expected; secondly, due to various factors such as hemodialysis patients being generally advanced in age, suffering from a variety of chronic diseases, limited health awareness, fear of side effects, and distrust of new things, hemodialysis patients are not very receptive to vaccines [55]. Although the response titer of vaccination in hemodialysis patients is low, it is undeniable that vaccination is still a powerful measure to prevent SARS-CoV-2 infection and serious consequences [56, 57].

According to the Chinese Center for Disease Control and Prevention, the overall mortality rate of SARS-CoV-2 is 2.3% [58]. It should be pointed out that the mortality rate of SARS-CoV-2 infection in patients receiving maintenance hemodialysis varies greatly in different periods or regions. During the study follow-up period in our center, 27 hemodialysis patients died, of which 8 patients died from non- SARS-CoV-2 infection causes. Our study ultimately reported an all-cause mortality rate of 5.2% (19/366) in patients with confirmed SARS-CoV-2 infection and a mortality rate of 2.7% (10/366) due to SARS-CoV-2 infection alone. Although the ICU admission rate among hemodialysis patients diagnosed with SARS-CoV-2 infection was 1.9%, the mortality rate was as high as 85.7% (6/7). During the diagnosis and treatment process in the dialysis center, it is recognized that not all patients diagnosed with infection need to be hospitalized. Elderly patients, patients with pulmonary or cardiovascular diseases, and patients with previous frailty can be arranged for hospitalization. In most cases, patients hospitalized in general wards Only nasal cannula or mask oxygen is required, and very few patients need to be admitted to the ICU and intubated [59]. During this period, not only our medical center, but most qualified medical centers are doing their best to open their wards to meet the demand for treatment. Thank you again for the efforts of all medical colleagues!

### Limitation

This article has several limitations. First, we performed PCR or antigen testing on every patient, but no laboratory sequence analysis of strain subtypes was performed. Due to the extremely scarce medical resources at that time, it was impossible to sequence the virus strain subtype for every patient. We followed reports investigated and published by the Chinese Center for Disease Control, which stated that during this period, all strains investigated were Omicron variants. Second, because this was a retrospective analysis, not all patients had all laboratory tests performed. Likewise, symptom and disease information, such as severe illness and death, was not collected for all cases. Finally, this was a single-center study. Our results represent only a specific patient population and do not extend to all patients infected with Omicron in other settings. Because the number of dialysis patients who received all three doses of the vaccine was too small, this may limit our further assessment of the impact of vaccination on survival, so the results need to be confirmed in other settings or prospective studies.

## Conclusions

In summary, patients receiving maintenance hemodialysis are at high risk for infection with the novel coronavirus. Our research points out that the mortality rate of hemodialysis patients infected with SARS-CoV-2 is higher than that of the general population. Our research report emphasizes that not only age is an independent risk factor for death, but also ensuring the adequacy of hemodialysis treatment and maintaining patients’ good physical condition are top priorities. It is crucial to ensure regular dialysis treatment for hemodialysis patients, even during the SARS-CoV-2 outbreak. It is unwise to stop dialysis because of fear of infection, which may cause more complications and lead to dangerous outcomes. Through this retrospective study, this article puts forward the above points for communication in anticipation of the need to re-evaluate public health and social measures to deal with any more transmissible SARS-CoV-2 variants in the future.

## Data Availability

To protect patient privacy, the data in this article have not been made public. But are available from the corresponding author on reasonable request.

## References

[1] Wu JT, Leung K, Leung GM (2020). Nowcasting and forecasting the potential domestic and international spread of the 2019-nCoV outbreak originating in Wuhan, China: a modelling study. Lancet.

[2] Fan Y, Li X, Zhang L, Wan S, Zhang L, Zhou F (2022). SARS-CoV-2 Omicron variant: recent progress and future perspectives. Signal Transduct Target Ther.

[3] CHINESE CENTER FOR DISEASE CONTROL AND PREVENTION. National situation of novel coronavirus infection https://www.chinacdc.cn/jkzt/crb/zl/szkb_11803/jszl_13141/202301/t20230125_263519.html. Accessed 12 Nov 2023.

[4] Collaboration GBDCKD (2020). Global, regional, and national burden of chronic kidney disease, 1990–2017: a systematic analysis for the global burden of Disease Study 2017. Lancet.

[5] Xiong F, Tang H, Liu L, Tu C, Tian JB, Lei CT, Liu J, Dong JW, Chen WL, Wang XH (2020). Clinical characteristics of and medical interventions for COVID-19 in Hemodialysis patients in Wuhan, China. J Am Soc Nephrol.

[6] Hsu CM, Weiner DE, Aweh G, Miskulin DC, Manley HJ, Stewart C, Ladik V, Hosford J, Lacson EC, Johnson DS (2021). COVID-19 among US Dialysis patients: risk factors and outcomes from a National Dialysis Provider. Am J Kidney Dis.

[7] Carlson N, Nelveg-Kristensen KE, Freese Ballegaard E, Feldt-Rasmussen B, Hornum M, Kamper AL, Gislason G, Torp-Pedersen C (2021). Increased vulnerability to COVID-19 in chronic kidney disease. J Intern Med.

[8] Chang CH, Fan PC, Kuo G, Lin YS, Tsai TY, Chang SW, Tian YC, Lee CC (2020). Infection in Advanced chronic kidney disease and subsequent adverse outcomes after Dialysis initiation: a Nationwide Cohort Study. Sci Rep.

[9] Chung EYM, Palmer SC, Natale P, Krishnan A, Cooper TE, Saglimbene VM, Ruospo M, Au E, Jayanti S, Liang A (2021). Incidence and outcomes of COVID-19 in people with CKD: a systematic review and Meta-analysis. Am J Kidney Dis.

[10] Council E-E, Group EW (2021). Chronic kidney disease is a key risk factor for severe COVID-19: a call to action by the ERA-EDTA. Nephrol Dial Transpl.

[11] NATIONAL HEALTH COMMISSION OF CHINA. New coronavirus pneumonia prevention and control program, 10th edn. In: https://www.gov.cn/zhengce/zhengceku/2023-01/06/5735343/files/5844ce04246b431dbd322d8ba10afb48.pdf. Accessed 12 Nov 2023.

[12] Kurts C, Panzer U, Anders HJ, Rees AJ (2013). The immune system and kidney disease: basic concepts and clinical implications. Nat Rev Immunol.

[13] Alfano G, Ferrari A, Magistroni R, Fontana F, Cappelli G, Basile C (2021). The frail world of haemodialysis patients in the COVID-19 pandemic era: a systematic scoping review. J Nephrol.

[14] Organization WH. WHO Coronavirus (COVID-19) Dashboard. 2023. https://covid19.who.int/. Accessed 12 Nov 2023.

[15] Chen N, Zhou M, Dong X, Qu J, Gong F, Han Y, Qiu Y, Wang J, Liu Y, Wei Y (2020). Epidemiological and clinical characteristics of 99 cases of 2019 novel coronavirus pneumonia in Wuhan, China: a descriptive study. Lancet.

[16] Wang D, Hu B, Hu C, Zhu F, Liu X, Zhang J, Wang B, Xiang H, Cheng Z, Xiong Y (2020). Clinical characteristics of 138 hospitalized patients with 2019 Novel Coronavirus-infected pneumonia in Wuhan, China. JAMA.

[17] Cheng Y, Luo R, Wang K, Zhang M, Wang Z, Dong L, Li J, Yao Y, Ge S, Xu G (2020). Kidney disease is associated with in-hospital death of patients with COVID-19. Kidney Int.

[18] Nugent J, Aklilu A, Yamamoto Y, Simonov M, Li F, Biswas A, Ghazi L, Greenberg H, Mansour G, Moledina G (2021). Assessment of Acute kidney Injury and longitudinal kidney function after hospital discharge among patients with and without COVID-19. JAMA Netw Open.

[19] Fang L, Karakiulakis G, Roth M (2020). Are patients with hypertension and diabetes mellitus at increased risk for COVID-19 infection?. Lancet Respiratory Med.

[20] Pecly IMD, Azevedo RB, Muxfeldt ES, Botelho BG, Albuquerque GG, Diniz PHP, Silva R, Rodrigues CIS (2021). COVID-19 and chronic kidney disease: a comprehensive review. Jornal brasileiro de nefrologia.

[21] Yang X, Yu Y, Xu J, Shu H, Xia J, Liu H, Wu Y, Zhang L, Yu Z, Fang M (2020). Clinical course and outcomes of critically ill patients with SARS-CoV-2 pneumonia in Wuhan, China: a single-centered, retrospective, observational study. Lancet Respiratory Med.

[22] Huang C, Wang Y, Li X, Ren L, Zhao J, Hu Y, Zhang L, Fan G, Xu J, Gu X (2020). Clinical features of patients infected with 2019 novel coronavirus in Wuhan, China. Lancet.

[23] Keller N, Chantrel F, Krummel T, Bazin-Kara D, Faller AL, Muller C, Nussbaumer T, Ismer M, Benmoussa A, Brahim-Bouna M (2020). Impact of first-wave COronaVIrus disease 2019 infection in patients on haemoDIALysis in Alsace: the observational COVIDIAL study. Nephrol Dial Transpl.

[24] Wang R, Liao C, He H, Hu C, Wei Z, Hong Z, Zhang C, Liao M, Shui H (2020). COVID-19 in Hemodialysis patients: a report of 5 cases. Am J Kidney Dis.

[25] Zheng XY, Zhang ZH, Cheng YM, Yang Q, Xu B, Lai BC, Huang LT (2023). Factors associated with subgroups of fatigue in maintenance hemodialysis patients: a cross-sectional study. Ren Fail.

[26] Wang SY, Zang XY, Fu SH, Bai J, Liu JD, Tian L, Feng YY, Zhao Y (2016). Factors related to fatigue in Chinese patients with end-stage renal disease receiving maintenance hemodialysis: a multi-center cross-sectional study. Ren Fail.

[27] Bonetti G, Manelli F, Patroni A, Bettinardi A, Borrelli G, Fiordalisi G, Marino A, Menolfi A, Saggini S, Volpi R (2020). Laboratory predictors of death from coronavirus disease 2019 (COVID-19) in the area of Valcamonica, Italy. Clin Chem Lab Med.

[28] Guan WJ, Ni ZY, Hu Y, Liang WH, Ou CQ, He JX, Liu L, Shan H, Lei CL, Hui DSC (2020). Clinical characteristics of Coronavirus Disease 2019 in China. N Engl J Med.

[29] Fan BE, Chong VCL, Chan SSW, Lim GH, Lim KGE, Tan GB, Mucheli SS, Kuperan P, Ong KH (2020). Hematologic parameters in patients with COVID-19 infection. Am J Hematol.

[30] Tian S, Hu W, Niu L, Liu H, Xu H, Xiao SY (2020). Pulmonary Pathology of Early-Phase 2019 Novel Coronavirus (COVID-19) pneumonia in two patients with Lung Cancer. J Thorac Oncology: Official Publication Int Association Study Lung Cancer.

[31] Xu Z, Shi L, Wang Y, Zhang J, Huang L, Zhang C, Liu S, Zhao P, Liu H, Zhu L (2020). Pathological findings of COVID-19 associated with acute respiratory distress syndrome. Lancet Respiratory Med.

[32] Jafarzadeh A, Jafarzadeh S, Nozari P, Mokhtari P, Nemati M (2021). Lymphopenia an important immunological abnormality in patients with COVID-19: possible mechanisms. Scand J Immunol.

[33] Liu J, Li S, Liu J, Liang B, Wang X, Wang H, Li W, Tong Q, Yi J, Zhao L (2020). Longitudinal characteristics of lymphocyte responses and cytokine profiles in the peripheral blood of SARS-CoV-2 infected patients. EBioMedicine.

[34] Kular D, Chis Ster I, Sarnowski A, Lioudaki E, Braide-Azikiwe DCB, Ford ML, Makanjuola D, Rankin A, Cairns H, Popoola J et al. The Characteristics, Dynamics, and the Risk of Death in COVID-19 Positive Dialysis Patients in London, UK. *Kidney360* 2020, 1(11):1226–1243. 10.34067/KID.0004502020 PMC8815514 35372882

[35] Kamel MH, Mahmoud H, Zhen A, Liu J, Bielick CG, Mostaghim A, Lin N, Chitalia V, Ilori T, Waikar SS (2021). End-stage kidney disease and COVID-19 in an urban safety-net hospital in Boston, Massachusetts. PLoS ONE.

[36] Fakhrolmobasheri M, Vakhshoori M, Heidarpour M, Najimi A, Mozafari AM, Rezvanian H. Hypophosphatemia in Coronavirus Disease 2019 (COVID-19), Complications, and Considerations: A Systematic Review. *BioMed research international* 2022, 2022:1468786. 10.1155/2022/1468786 PMC9616661 36312855

[37] Wang L, Xiao C, Chen L, Zhang X, Kou Q (2019). Impact of hypophosphatemia on outcome of patients in intensive care unit: a retrospective cohort study. BMC Anesthesiol.

[38] Mercola J, Grant WB, Wagner CL. Evidence regarding vitamin D and risk of COVID-19 and its severity. Nutrients 2020, 12(11). 10.3390/nu12113361 PMC7692080 33142828

[39] Bonnet JB, Berchoux E, Sultan A (2021). Decompensated primary hypoparathyroidism in a patient with COVID-19. Ann Endocrinol.

[40] Mabillard H, Sayer JA (2020). Electrolyte disturbances in SARS-CoV-2 infection. F1000Research.

[41] Boyd S, Sheng Loh K, Lynch J, Alrashed D, Muzzammil S, Marsh H, Masoud M, Bin Ihsan S, Martin-Loeches I. The incidence of venous thromboembolism in critically ill patients with SARS-CoV-2 infection compared with critically ill influenza and community-acquired pneumonia patients: a Retrospective Chart Review. Med Sci (Basel Switzerland) 2022, 10(2). 10.3390/medsci10020030 PMC9231025 35736350

[42] Zhang Y, Xiao M, Zhang S, Xia P, Cao W, Jiang W, Chen H, Ding X, Zhao H, Zhang H (2020). Coagulopathy and Antiphospholipid Antibodies in patients with Covid-19. N Engl J Med.

[43] Parks AL, Auerbach AD, Schnipper JL, Bertram A, Jeon SY, Boyle B, Fang MC, Gadrey SM, Siddiqui ZK, Brotman DJ (2022). Venous thromboembolism (VTE) prevention and diagnosis in COVID-19: practice patterns and outcomes at 33 hospitals. PLoS ONE.

[44] Khoo BZE, Lim RS, See YP, Yeo SC (2021). Dialysis circuit clotting in critically ill patients with COVID-19 infection. BMC Nephrol.

[45] Tang N, Li D, Wang X, Sun Z (2020). Abnormal coagulation parameters are associated with poor prognosis in patients with novel coronavirus pneumonia. J Thromb Haemostasis: JTH.

[46] Walther CP, Carter CW, Low CL, Williams P, Rifkin DE, Steiner RW, Ix JH (2012). Interdialytic creatinine change versus predialysis creatinine as indicators of nutritional status in maintenance hemodialysis. Nephrol Dial Transpl.

[47] Song H, Wei C, Hu H, Wan Q (2022). Association of the serum albumin level with prognosis in chronic kidney disease patients. Int Urol Nephrol.

[48] Pan W, Han Y, Hu H, He Y (2022). Association between hemoglobin and chronic kidney disease progression: a secondary analysis of a prospective cohort study in Japanese patients. BMC Nephrol.

[49] Wu J, Li W, Shi X, Chen Z, Jiang B, Liu J, Wang D, Liu C, Meng Y, Cui L (2020). Early antiviral treatment contributes to alleviate the severity and improve the prognosis of patients with novel coronavirus disease (COVID-19). J Intern Med.

[50] Cantarelli C, Angeletti A, Perin L, Russo LS, Sabiu G, Podestà MA, Cravedi P (2022). Immune responses to SARS-CoV-2 in dialysis and kidney transplantation. Clin Kidney J.

[51] Van Praet J, Reynders M, De Bacquer D, Viaene L, Schoutteten MK, Caluwé R, Doubel P, Heylen L, De Bel AV, Van Vlem B (2021). Predictors and dynamics of the Humoral and Cellular Immune response to SARS-CoV-2 mRNA vaccines in Hemodialysis patients: a Multicenter Observational Study. J Am Soc Nephrol.

[52] Espi M, Charmetant X, Barba T, Koppe L, Pelletier C, Kalbacher E, Chalencon E, Mathias V, Ovize A, Cart-Tanneur E (2021). The ROMANOV study found impaired humoral and cellular immune responses to SARS-CoV-2 mRNA vaccine in virus-unexposed patients receiving maintenance hemodialysis. Kidney Int.

[53] Stumpf J, Siepmann T, Lindner T, Karger C, Schwöbel J, Anders L, Faulhaber-Walter R, Schewe J, Martin H, Schirutschke H (2021). Humoral and cellular immunity to SARS-CoV-2 vaccination in renal transplant versus dialysis patients: a prospective, multicenter observational study using mRNA-1273 or BNT162b2 mRNA vaccine. Lancet Reg Health Europe.

[54] Karakizlis H, Nahrgang C, Strecker K, Chen J, Aly M, Slanina H, Schüttler CG, Esso I, Wolter M, Todorova D (2022). Immunogenicity and reactogenicity of homologous mRNA-based and vector-based SARS-CoV-2 vaccine regimens in patients receiving maintenance dialysis. Clin Immunol (Orlando Fla).

[55] Xiao J, Cheung JK, Wu P, Ni MY, Cowling BJ, Liao Q (2022). Temporal changes in factors associated with COVID-19 vaccine hesitancy and uptake among adults in Hong Kong: serial cross-sectional surveys. Lancet Reg Health Western Pac.

[56] Hsu CM, Weiner DE, Manley HJ, Aweh GN, Ladik V, Frament J, Miskulin D, Argyropoulos C, Abreo K, Chin A (2022). Seroresponse to SARS-CoV-2 vaccines among maintenance Dialysis patients over 6 months. Clin J Am Soc Nephrology: CJASN.

[57] Oliver MJ, Thomas D, Balamchi S, Ip J, Naylor K, Dixon SN, McArthur E, Kwong J, Perl J, Atiquzzaman M (2022). Vaccine effectiveness against SARS-CoV-2 infection and severe outcomes in the Maintenance Dialysis Population in Ontario, Canada. J Am Soc Nephrol.

[58] Wu Z, McGoogan JM (2020). Characteristics of and important lessons from the Coronavirus Disease 2019 (COVID-19) outbreak in China: Summary of a report of 72 314 cases from the Chinese Center for Disease Control and Prevention. JAMA.

[59] Alfano G, Morisi N, Frisina M, Ferrari A, Fontana F, Tonelli R, Franceschini E, Meschiari M, Donati G, Guaraldi G (2022). Awaiting a cure for COVID-19: therapeutic approach in patients with different severity levels of COVID-19. Le Infezioni in Medicina.

